# PD-1 inhibitors-based second-line therapy for metastatic gastric cancer

**DOI:** 10.3389/fimmu.2023.1136437

**Published:** 2023-05-22

**Authors:** Miaomiao Gou, Yong Zhang, Zhikuan Wang, Guanghai Dai

**Affiliations:** ^1^ Medical Oncology Department, The Fifth Medical Center, Chinese People’s Liberation Army General Hospital, Beijing, China; ^2^ Medical Oncology Department, The Second Medical Center, Chinese People’s Liberation Army General Hospital, Beijing, China

**Keywords:** PD-1 inhibitors, second-line therapy, metastatic gastric cancer, angiogenesis, immunotherapy

## Abstract

**Background:**

Metastatic gastric cancer (MGC) patients with progression on first-line treatment still have poor outcomes on chemotherapy. The KEYNOTE-061 study demonstrated that pembrolizumab, a PD-1inhibitor, was not better than paclitaxel as second-line therapy for MGC. Herein, we explored the efficacy and safety of PD-1inhibitor based treatment for MGC patients in the second line.

**Methods:**

In this observational, retrospective study, we enrolled MGC patients treated with anti-PD-1 based therapy as second-line in our hospital. We primarily assessed the treatment’s efficacy and safety. We also evaluated the relationship between clinical features and outcomes using univariate and multivariate analyses.

**Results:**

We enrolled 129 patients with an objective response rate (ORR) of 16.3% and a disease control rate (DCR) of 79.1%. Patients treated with PD-1inhibitor combined with chemotherapy and anti-angiogenic agents had ORR of 19.6% and higher DCR of 94.1%. The median progression-free survival (PFS) was 4.10 months, and the median overall survival (OS) was 7.60 months. In univariate analysis, patients treated with PD-1inhibitor combined with chemotherapy and anti-angiogenic agents and with prior anti-PD-1 history were significantly associated with favorable PFS and OS. In the multivariate analysis, different combination therapy and prior anti-PD-1 history were independent prognosis biomarkers for PFS and OS. Grade 3 or 4 treatment-related adverse events (TRAEs) occurred in 28 (21.7%) patients. Common adverse events (AEs) included fatigue, hyper/hypothyroidism, neutrophil decrease, anemia, skin reactions, proteinuria, and hypertension. We did not observe treatment-related deaths.

**Conclusion:**

Our current results indicated that PD-1-inhibitor and chemo-anti-angiogenic agents combination therapy and prior PD-1 treatment history might improve clinical activity for GC immunotherapy as second-line treatment with acceptable safety profiles. Further studies are needed to verify those outcomes for MGC in other centers.

## Introduction

Gastric cancer (GC) is the most common malignant tumor with heterogeneous diseases (1) and the second cancer-related death cause in China (2). The treatment of GC patients is currently suboptimal due to underlying tumor molecular biology. Before chemotherapy, the overall survival (OS) of advanced or metastatic GC (AGC or MGC) was less than one year (3). The advent of checkpoint inhibitors led to a fundamental change in treating some tumors. Immunotherapy, especially anti-PD-1(programmed death-1) therapy, has shown promising anticancer first-line activity for lung cancer (4), melanoma (5), esophagogastric cancer (6), and metastatic colorectal carcinoma harboring mismatch repair-deficient (dMMR) or microsatellite instability-high (MSI-H) phenotypes (7). In the 2020 ESMO meeting, Checkmate-649 (8) and ATTRCATION-4 (9) results promoted a debate that has lasted until now. Both trials demonstrated superior efficacy and progression-free survival (PFS) with PD-1inhibitor combined with chemotherapy to traditional first-line 5-Fuor Oxaliplatin-based treatment for her-2 negative MGC.

However, second-line treatments for MGC remain poor. The survival with treatments, such as cytotoxic chemotherapy with docetaxel, paclitaxel, or irinotecan, is less than six months (10, 11). Based on the observed response in the REGARD and RAINBOW study (12, 13), the US Food and Drug Administration (FDA) granted the use of ramucirumab(an anti-vascular endothelial growth factor receptor-2 agent, anti-VEGFR-2) with paclitaxel or ramucirumab monotherapy for recurrent locally advanced or metastatic GC who progressed on or after two or more previous lines of therapy. However, the OS of these treatments is still limited. Moreover, the first-time use of immunotherapy in the second line failed the KEYNOTE-061 study with pembrolizumab, a PD-1inhibitor, which did not significantly improve OS compared to paclitaxel as second-line therapy for MGC with PD-L1 (programmed death ligand-1) CPS of one or higher (14). This result triggered a quest to find new systemic combinations to improve MGC patients’ outcomes.

Anti-angiogenic therapies had provided hope for patients with later-stage gastric cancer in the second or further line based on several studies. In exception of ramucirumab we mentioned above, Apatinib, a selective VEGFR-2 tyrosine kinase inhibitor was also shown to prolong overall survival as a third-line or later treatment option in patients with advanced gastric cancer (15). A few studies with very limited cases have reported anticancer activities when checkpoint inhibitors are combined with anti-angiogenic drugs (16–18). To the best of our knowledge, little research has summarized the results of PD-1inhibitors combined other kinds of agents as second-line therapy for MGC in different situation. Therefore, in the present study, we retrospectively analyzed the outcomes of her-2 negative late-stage GC patients treated with PD-1inhibitors based therapy as second-line.

## Materials and methods

### Study population

We retrospectively collected the medical records from a series of consecutive AGC or MGC patients with a PD-1inhibitor (immune checkpoint inhibitor - ICI) as second-line treatment at the Oncology Department of Chinese PLA General Hospital between October 1, 2015, and August 31, 2022. The selection criteria included: confirmed pathologic diagnosis as adenocarcinoma GC; had at least one measurable lesion; received at least two cycles of anti-PD-1inhibitors as second-line therapy; and had imaging response assessment through the treatment period and time of survival. The major exclusion criteria included: patients with MMR-deficient tumors who received anti-PD-L1 antibodies without efficacy evaluation. We retrieved and integrated clinical and basic features from enrolled patients’ records, including age, gender, PD-L1 expression status, Eastern Cooperative Oncology Group Performance Status (ECOG PS), tumor location, histological differentiation, metastasis organs, smoking and drinking habit, surgery history, prior immunotherapy status, and treatment regime.

This study was approved by the Ethics Committee of the Chinese PLA General Hospital (No. S2019-200-01) and was conducted following the Declaration of Helsinki. All patients signed informed consent forms for treatment. Due to the retrospective nature of this study, the Ethics Committee of Chinese PLA General Hospital did not require further consent from the patients to use their medical records.

### Study design and treatment regimens

This was a retrospective, single-center study. According to the patient’s records, an anti-PD-1inhibitor was administered in combination with chemotherapy or anti-angiogenic agents in the second line. Patients received 200 mg pembrolizumab, nivolumab at 3 mg/kg, 200 mg sintilimab, 200 mg carrelizumab, or 240 mg toripalimab intravenously once every three weeks after chemo or other agents. Combined chemotherapy included Paclitaxel or Irinotecan, Oxaliplatin and other combinations. Besides chemotherapy, small-molecule tyrosine kinase inhibitors (TKI) (Apatinib) and monoclonal antibodies, such as Bevacizumab, were used as combined anti-angiogenic agents.

### Assessment

We primarily evaluated the efficacy and safety of PD-1inhibitor-based therapy as second line in advanced MGC patients. According to Response Evaluation Criteria in Solid Tumors 1.1, the efficacy of immunotherapy includes complete response (CR), partial response (PR), stable disease (SD), and progressive disease (PD). The percentage of patients who achieved a CR or PR comprises the objective response rate (ORR), and the percentage of patients who achieved a CR, PR, or SD represents the disease control rate (DCR). The OS is the time from treatment to death, and the PFS is the time from treatment to the first occurrence of PD or death. We evaluated adverse events (AEs) using the National Cancer Institute Common Terminology Criteria for Adverse Events (version 4.03) and the Common Terminology Criteria for Adverse Events (version 4.0). The cut-off date was August 31, 2021.

### Statistical analysis

Baseline characteristics are presented as proportions for categorical variables and medians for continuous variables. Categorical data comparisons were conducted using Pearson’s χ^2^ or Fisher’s exact test. The Kaplan–Meier method was used to analyze survival data. The Log-rank test and Cox proportional hazard regression were used to examine the relationship between clinical features, OS, and PFS. Statistical analyses were conducted using SPSS 21.0 (SPSS Inc., Chicago, IL, USA). A *p* < 0.05 was considered statistically significant. R-studio (version 1.03) was used to plot the Kaplan–Meier curve.

## Results

### Patients and tumor characteristics

We included 129 patients with microsatellite stable type (MSS) tumors, with a median age of 60 years (range: 33–86). Of these patients, 70.5% patients were male and 81.4% (105 out of 129) had an ECOG performance status of 0-1. PD-L1 expression was negative in 65.1% (84 out of 129) of patients, while 43.4% (56 out of 129) had multiple metastatic lesions (> 2), and 40.3% (53 out of 129) had liver metastasis. Among the patients, 50.4% (65 out of 129) had undergone surgery. 24% of the patients had received immunotherapy in first-line treatment. The PD-1 inhibitors received by the patients included carrelizumab (n = 33), sinitinib (n = 38), pembrolizumab (n = 18), toripalimab (n = 13), and nivolumab (n = 27). Seventy-eight patients received PD-1 inhibitor plus chemotherapy, and 51 patients received chemotherapy and anti-angiogenic agent drugs combined with a PD-1 inhibitor as second-line treatment. Clinical features in above two different treatment groups were well balanced. All patients had MMR-proficient tumors. **Table 1** shows all the clinical variables.

**Table 1:** Baseline characteristics of patients (n=129).

Characteristics	No. patients	%	Combination therapy		P value
			Plus chemo	Plus chemo+ anti-angiogenic agents	
Total	129				
Gender					
Male	91	70.5%	54	37	0.686
Female	38	29.5%	24	14	
"Age					
<=60	67	51.9%	43	24	0.370
>60	62	48.1%	35	27	
ECOG PS					
0-1	105	81.4%	64	41	0.813
>=2	24	18.6%	14	10	
Tumor location					
Cardia	31	24.0%	16	15	0.491
Body/Fundus	40	31.0%	26	14	
Pylorus	58	45.0%	36	22	
Histological differentiation					
Poorly	72	55.8%	43	29	0.366
Moderately	54	41.9%	32	22	
Well	3	2.3%	3	0	
PD-L1 expression					
Negative	84	65.1%	53	31	0.404
Positive	45	34.9%	25	20	
Number of metastatic organs					
<=2	73	56.6%	45	28	0.755
>2	56	43.4%	33	23	
Liver metastasis					
Yes	53	41.1%	31	22	0.702
No	76	58.9%	47	29	
Smoking history					
Yes	52	40.3%	35	17	0.191
No	77	59.7%	43	34	
Drinking history					
Yes	56	43.4%	32	24	0.499
No	73	56.6%	46	27	
Prior surgery					
Yes	65	50.4%	37	28	0.407
No	64	49.6%	41	23	
Immunotherapy in first line					
Yes	31	24.0%	17	14	0.462
No	98	76.0%	61	37	
PD-1 combination therapy					
Plus chemo	78	60.5%	/	/	/
Plus chemo+anti-angiogenic agents	51	39.5%	/	/	/
anti-PD-1 type					
carrelizumab	33	25.0%	/	/	/
sinitinib	38	28.9%	/	/	/
pembrolizumab	18	14.4%	/	/	/
toripalimab	13	9.8%	/	/	/
nivolumab	27	21.9%	/	/	/
Chemotherapy					
Oxaliplatin based	21	16.3%	/	/	/
Paclitaxel-like based	83	64.3%	/	/	/
Irinotecan based	25	19.4%	/	/	/
anti-angiogenic agents					
Apatinib	48	94.1%	/	/	/
Bevacizumab	3	5.9%	/	/	/

ECOG PS, Eastern Cooperative Oncology Group Performance Status; PD-L1, programmed death ligand-1. Plus chemo, plus chemotherapy. Plus chemo+anti-angiogenic agents, Plus chemotherapy plus anti-angiogenic agents.

### Efficacy

Response to treatment was analyzed in all 129 patients. Twenty-one (16.3%) patients presented ORR with two CRs. Additionally, 79.1% (n = 102) of patients achieved disease control (**Table 2**). The ORR and DCR for patient subgroups are shown in **Table 2**. The ORR and DCR did not differ between PD-L1 expression subgroups (P>0.05). Patients who had received PD-1 inhibitors in the first line and received PD-1 inhibitor combined with chemotherapy and anti-angiogenic agents had ORR of 22.6% and 19.6%, respectively. DCR of patients in the PD-1 inhibitor combined chemotherapy and anti-angiogenic agents groups was higher when compared with the counterpart group (94.1% vs. 69.2%, P=0.001).

**Table 2:** Response outcome.

Response	No. patients	%	P
Total	129		
ORR (CR+PR), %	**16.3%**		
PD-L1 expression			
Negative	17/84	20.2%	0.096
Positive	4/45	8.9%	
Immunotherapy in first line			
Yes	7/31	22.6%	0.276
No	14/98	14.3%	
PD-1 combination therapy			
Plus chemo	11/78	14.1%	0.408
Plus chemo+anti-angiogenic agents	10/51	19.6%	
DCR (CR+PR+SD), %	**79.1%**		
PD-L1 expression			
Negative	67/84	79.8%	0.792
Positive	35/45	77.8%	
Immunotherapy in first line			
Yes	27/31	87.1%	0.208
No	75/98	76.5%	
PD-1 combination therapy			
Plus chemo	54/78	69.2%	0.001
Plus chemo+anti-angiogenic agents	48/51	94.1%	

**Figure 1:**
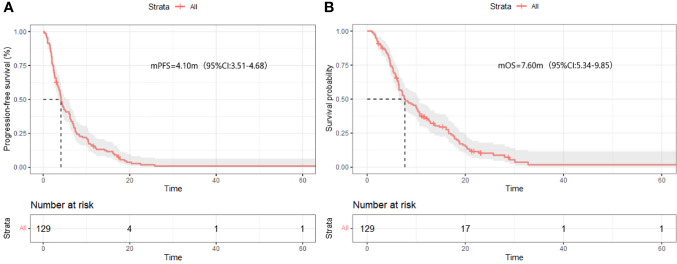
Progression-free survival (PFS) **(A)** and Overall survival (OS) **(B)** in all patients.

**Figure 2:**
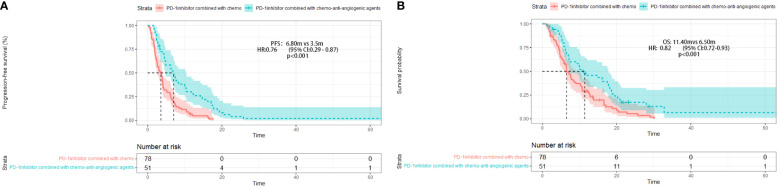
Progression-free survival (PFS) **(A)** and Overall survival (OS) **(B)** in different treatment group.

**Figure 3:**
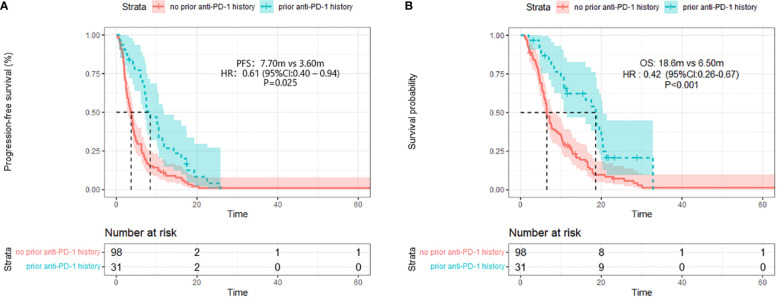
Progression-free survival (PFS) **(A)** and Overall survival **(B)** (right) in different prior PD-1 treatment history group.

After four months of follow-up, the median PFS was 4.10 months [95% confidence interval (CI): 3.51-4.68; **Figure 1A**, **Table 3**]. At the cut-off date, six patients were still alive. The median OS was 7.60 months [95% CI: 5.34-9.85; **Figure 1B**, **Table 3**]. In the univariate analysis, the treatment effect was greater for both OS and PFS in patients treated with PD-1 inhibitors combined with chemotherapy and anti-angiogenic agents and those with prior anti-PD-1 history (**Tables 4**, **5**). The median OS was 11.40 months for patients receiving PD-1 inhibitor and chemo plus anti-angiogenic agents treatment versus 6.50 months for patients with combined with chemo [hazard ratio (HR): 0.82, 95% CI: 0.72-0.93; p<0.001, **Figure 2B**, **Table 3**]. Patients with first-line PD-1 inhibitors had significantly prolonged OS compared to those without (18.60 vs. 6.50 months, HR: 0.42, 95% CI: 0.26-0.67; p < 0.001, **Figure 3B**, **Table 3**). The median PFS was 6.80months (95% CI, 4.58-9.01) in patients treated with PD-1-chemo-anti-angiogenic agents and 3.50 months (95% CI, 2.47-4.52) in patients with PD-1 combined with chemotherapy (**Figure 2A**, **Table 3**). Patients with prior anti-PD-1 administration had a higher PFS months in contrast to those with no prior anti-PD-1 history (7.70 vs 3.50months, P=0.025) (**Figure 3A**, **Table 3**). Meanwhile, the multivariate analysis showed that different combination therapy and prior anti-PD-1 history were independent prognostic biomarkers for PFS and OS (**Tables 4**, **5**).

**Table 3:** Survival of different clinical groups.

Survival	Median months (95%CI)	Log Rank P
PFS, median (95% CI)	4.10 (3.51 - 4.68)	
PD-L1 expression		
Negative	4.10 (3.36 - 4.83)	0.455
Positive	3.90 (3.39 - 4.40)	
Immunotherapy in first line		
Yes	7.70 (4.66 - 10.73)	0.025
No	3.50 (2.80 - 4.39)	
PD-1 combination therapy		
Plus chemo	3.50 (2.47 - 4.52)	0.000
Plus chemo+anti-angiogenic agents	6.80 (4.58 - 9.01)	
OS, median (95% CI)	7.60m (5.34-9.85)	
PD-L1 expression		
Negative	7.60 (5.65 - 9.54)	0.227
Positive	7.60 (4.37 - 10.82)	
Immunotherapy in first line		
Yes	18.60 (15.52 - 21.67)	0.000
No	6.50 (5.74 - 7.25)	
PD-1 combination therapy		
Plus chemo	6.50 (5.33 - 7.67)	0.000
Plus chemo+anti-angiogenic agents	11.40 (5.50 - 17.30)	

**Table 4:** Univariate analysis and multivariate analysis of clinical variables for the prediction of progression free survival.

Variable		Univariate analysis		Multivariate analysis	
		HR	P	HR	P
Gender	Female vs Male	1.24 (0.84 - 1.844)	0.268	0.93 (0.57 - 1.50)	0.773
Age	>60 vs <=60	0.90 (0.63 - 1.296)	0.594	0.93 (0.64 - 1.34)	0.703
ECOG PS	>=2 vs 0-1	1.43(0.91 - 2.247)	0.117	1.57(0.95 - 2.59)	0.073
Tumor location	Pylorus vs Body/Fundus vs Cardia	1.05 (0.84 - 1.310)	0.666	0.97 (0.77 - 1.23)	0.827
Histological differentiation	Well vs Moderately vs Poorly	0.93 (0.66 - 1.324)	0.721	1.00 (0.68 - 1.45)	0.998
PD-L1 expression	Positive vs Negative	1.15 (0.79 - 1.679)	0.460	1.03 (0.69 - 1.54)	0.877
No.of metastasis organs	>2 vs <=2	1.06(0.74 - 1.520)	0.745	1.05 (0.71 - 1.54)	0.787
Liver metastasis	No vs Yes	1.07 (0.74 - 1.536)	0.708	1.23 (0.80 - 1.89)	0.328
Smoking history	No vs Yes	1.17 (0.81 - 1.683)	0.389	1.21 (0.75 - 1.96)	0.423
Drinking history	No vs Yes	1.35 (0.94 - 1.941)	0.101	1.09 (0.65 - 1.83)	0.730
Prior surgery	No vs Yes	1.23 (0.86 - 1.776)	0.243	1.00 (0.65 - 1.53)	0.990
Immunotherapy in first line	Yes vs No	0.61 (0.40 – 0.94)	0.025	0.61(0.38 – 0.99)	0.046
PD-1 combination therapy	Plus chemo+anti-angiogenic agents vs Plus chemo	0.76 (0.29 - 0.87)	0.000	0.75 (0.65 - 0.86)	0.000

**Table 5:** Univariate analysis and multivariate analysis of clinical variables for the prediction of overall survival.

Variable		Univariate analysis		Multivariate analysis	
		HR	P	HR	P
Gender	Female vs Male	1.10 (0.73 - 1.65)	0.647	0.94 (0.58 - 1.52)	0.814
Age	>60 vs <=60	1.06(0.73 - 1.54)	0.753	1.10 (0.73 - 1.64)	0.635
ECOG PS	>=2 vs 0-1	1.20 (0.76 - 1.91)	0.419	1.26 (0.77 - 2.08)	0.350
Tumor location	Pylorus vs Body/Fundus vs Cardia	0.84 (0.66 - 1.06)	0.158	0.77 (0.60 - 0.99)	0.045
Histological differentiation	Well vs Moderately vs Poorly	0.79 (0.55 - 1.13)	0.209	0.75 (0.50 - 1.11)	0.154
PD-L1 expression	Positive vs Negative	1.27 (0.85 - 1.90)	0.232	1.14 (0.74 - 1.74)	0.534
No.of metastasis organs	>2 vs <=2	1.00 (0.69 - 1.45)	0.982	0.88 (0.59 - 1.31)	0.545
Liver metastasis	No vs Yes	1.12 (0.76 - 1.66)	0.544	1.19(0.77 - 1.84)	0.421
Smoking history	No vs Yes	0.91 (0.62 - 1.32)	0.621	0.94 (0.58 - 1.54)	0.829
Drinking history	No vs Yes	1.18 (0.81 - 1.72)	0.383	1.05 (0.62 - 1.80)	0.836
Prior surgery	No vs Yes	1.26 (0.87 - 1.84)	0.217	1.11 (0.71 - 1.75)	0.634
Immunotherapy in first line	Yes vs No	0.42 (0.26 – 0.67)	0.000	0.53 (0.32 – 0.87)	0.012
PD-1 combination therapy	Plus chemo+anti-angiogenic agents vs Plus chemo	0.82(0.72 - 0.93)	0.000	0.822 (0.71 - 0.94)	0.006

### Safety

Treatment-related adverse events (TRAEs) were observed in 110 (85.2%) out of 129 patients (**Table 6**). The most common TRAEs of any grade were fatigue (39.5%), hyper/hypothyroidism (29.4%), decreased neutrophil count (28.6%), anemia (26.3%), skin reactions (25.5%), proteinuria (20.1%), and hypertension (20.1%). Grade 3 TRAEs occurred in 26 (21.2%) patients and included hypertension [6 (4.6%)], anemia [5 (3.8%)], fatigue [5 (3.8%)], and skin reactions [5 (3.8%)]. No treatment-related deaths were reported, although one patient experienced intestinal perforation. TRAEs led to treatment discontinuation in five patients and dose reduction in 26 (20.1%) patients.

**Table 6:** Treatment-related adverse events.

	G1-2		G3-4	
Neutrophil decreased	37	28.6%	4	3.1%
PLT decreased	14	10.8%	2	1.5%
Anemia	34	26.3%	5	3.8%
Alanine/Aspartate aminotransferase increased	13	10.0%	1	0.7%
Bilirubin increased	11	8.5%	1	0.7%
Fatigue	51	39.5%	5	3.8%
Nausea	24	18.6%	5	3.8%
Hypertension	26	20.1%	6	4.6%
Skin reactions	33	25.5%	5	3.8%
Proteinuria	26	20.1%	4	3.1%
Diarrhoea	6	4.6%	1	0.7%
Hyper/Hypo thyroidism	38	29.4%	0	0.0%
Pneumonitis	10	7.7%	0	0.0%
occult blood postive	11	8.5%	0	0.0%

## Discussion

Immunotherapy has become first-and third-line treatment for MGC, according to Checkmate649 (19) and Attraction2 (20) studies. The failure of pembrolizumab in the KEYNOTE-061 (14) trial made it difficult for immunotherapy to become a second-line treatment, especially for patients who collapsed in the chemo-induced first line. Pembrolizumab did not significantly improve the OS compared to paclitaxel as second-line therapy for advanced gastric or gastroesophageal junction cancer with PD-L1 CPS of one or higher in the KEYNOTE-061. However, besides the favorable safety profile, the KEYNOTE-061 data supported further exploration to develop and identify the optional combination of pembrolizumab with other therapy regimens.

In our retrospective study, the overall ORR was 16.3%, and the DCR was 79.1% among the 129 patients. The ORR after PD-1-chemo- anti-angiogenic agents therapy or prior anti-PD-1history was 19.6% or 22.6%. In phase 3 of the KEYNOTE-061 trial, the ORRs of pembrolizumab as second-line treatment were 16% in patients with CPS of at least one and 2% in those with CPS of less than one. We found a significant increase in tumor response for anti-PD-1with anti-angiogenic agents among other studies. In a phase I/II study, the ORR was 37.2% in the 53 patients treated with nivolumab (NIVO) with paclitaxel (PTX) plus ramucirumab (RAM) (17). Meanwhile, the ORR was 26.3% (5/19), and the DCR was 63.2% (12/19) when patients received combination therapy of PD-1inhibitor and apatinib (21). Furthermore, lenvatinib plus pembrolizumab for AGC patients as first-line or second-line treatments presented an objective response in 69% of patients in the EPOC1706 study (18). In the current study, we observed a high ORR and DCR in the PD-1-chemo-anti-angiogenic agents group. The underlying mechanisms might be related to tumor anti-angiogenic agents inhibiting the extravasation of reactive T cells, which form an immunosuppressive microenvironment that leads to tumors escaping immunosurveillance (22, 23). Combination therapy strengthens T-cell infiltration and activation to eliminate tumor cells (24). These findings suggested that anti-angiogenic agents might overcome the resistance to PD-1monotherapy in the second line, which requires validation in a future clinical trial.

Herein, the survival data indicated that the anti-tumor effects of combination agents remain important as a second-line choice. Improvements in PFS (6.80months) and OS (11.40months) of the anti-PD-1blockade plus chemo-anti-angiogenic agents group indicated a favorable association. The same trend was detected for the first-line immunotherapy group, presenting a PFS of 7.70 months and OS of 18.60 months. Until now, the PFS of immunotherapy combined with anti-angiogenic agents is among 3.0-7.1 months as second-line therapy. Additionally, the median PFS is 3.0 (95% CI: 1.3-4.7) months for PD-1inhibitor combined with apatinib in unresectable locally advanced or metastatic GC patients (21). The median PFS was higher than 7.1 months in the EPOC1706 study for lenvatinib plus pembrolizumab treatment in AGC patients in the first and second lines (18). In contrast to previous studies, we found that treatment combination benefits and improves the efficacy of PD-1monotherapy or chemotherapy alone (14). Altogether, these data indicated that the optimal regimen impacts improved efficacy, emphasizing the necessity of changing treatment from PD-1monotherapy to chemo-anti-angiogenic combination.

We found a similar safety profile to other studies (14, 18, 21). No ever-reported AE occurred. The common AEs included fatigue, hyper/hypothyroidism, neutrophil decreases, anemia, skin reactions, proteinuria, and hypertension. Some AEs were anti-angiogenic agents related and manageable. One patient had intestinal perforation, which might be unrelated to the treatment (PD-1inhibitor plus docetaxel). Nevertheless, attention should be given to events such as pneumonitis and occult blood, which occurred in 7.7% and 8.5% of patients, respectively. Overall, combination therapy was safe and reliable for clinical application.

However, our current study also has some limitations. First, this was a single-center study with a limited population. Thus, further studies are required to investigate the efficacy of anti-PD-1combination treatment for MGC patients in other centers. Moreover, the lack of data, including EBER and tumor mutation burden, would have been related to the efficacy of immunotherapy.

## Conclusion

In summary, we found that PD-1inhibitors and anti-angiogenic agents combination therapy and prior PD-1 treatment history might improve the clinical activity of immunotherapy in GC as a second-line strategy. Hence, we provided a rationale for combining PD-1inhibitors and anti-angiogenic agents and chemotherapy after the failure of first-line therapy in late-stage GC.

## Data availability statement

The original contributions presented in the study are included in the article/supplementary material. Further inquiries can be directed to the corresponding author.

## Ethics statement

The studies involving human participants were reviewed and approved by the Ethics Committee of the Chinese PLA General Hospital. Written informed consent for participation was not required for this study in accordance with the national legislation and the institutional requirements.

## Author contributions

MG take charge in writing. YZ is responsible for data analyzing. ZW and GD are supervision and data collection. All authors contributed to the article and approved the submitted version.
